# An Efficient and Reliable Geographic Routing Protocol Based on Partial Network Coding for Underwater Sensor Networks

**DOI:** 10.3390/s150612720

**Published:** 2015-05-28

**Authors:** Kun Hao, Zhigang Jin, Haifeng Shen, Ying Wang

**Affiliations:** 1 School of Computer and Information Engineering, Tianjin ChengJian University, 300384 Tianjin, China; 2 School of Electronic Information Engineering, Tianjin University, 300072 Tianjin, China; E-Mail: zgjin@tju.edu.cn; 3 School of Computer Science, Engineering and Mathematics, Flinders University, 5001 Adelaide, Australia; E-Mail: haifeng.shen@flinders.edu.au

**Keywords:** underwater sensor networks (UWSNs), geographic routing, partial network coding, packet delivery ratio, energy consumption

## Abstract

Efficient routing protocols for data packet delivery are crucial to underwater sensor networks (UWSNs). However, communication in UWSNs is a challenging task because of the characteristics of the acoustic channel. Network coding is a promising technique for efficient data packet delivery thanks to the broadcast nature of acoustic channels and the relatively high computation capabilities of the sensor nodes. In this work, we present GPNC, a novel geographic routing protocol for UWSNs that incorporates partial network coding to encode data packets and uses sensor nodes’ location information to greedily forward data packets to sink nodes. GPNC can effectively reduce network delays and retransmissions of redundant packets causing additional network energy consumption. Simulation results show that GPNC can significantly improve network throughput and packet delivery ratio, while reducing energy consumption and network latency when compared with other routing protocols.

## 1. Introduction

In recently years, underwater sensor networks (UWSNs) [1,2] have been increasingly used in applications such as environmental monitoring, gas deposit exploration and exploitation, oceanographic data collection, oil spill monitoring, real-time warship monitoring, and disaster prevention. Although in general UWSNs are closely related to Wireless Sensor Networks (WSNs), several studies [3,4,5,6] have shown that many of the traditional techniques designed for WSNs are not applicable to UWSNs. It is the characteristics of the underwater channel—commonly regarded as one of the most difficult wireless communication channels—that make the design of efficient routing protocols for UWSNs a very challenging task [7]. Key issues with the underwater channel include high propagation latency due to the low speed of acoustic signals in water (typically 1500 m/s), severely limited available bandwidth, high noise, and high error rates. These issues lead to excessive data retransmissions, high energy consumption and low packet delivery ratios, which all contribute to the difficulties of designing an efficient and reliable routing protocol for UWSNs.

Geographic information routing has been widely accepted as a preferred method for routing packets in UWSNs as it does not require establishing/keeping complete routes or transmitting routing messages [8]. Each node knows its own location and the geographic information of the destination node and consequently can forward data packets to a locally optimal next-hop node closest to the destination node. As a result, geographic routing protocols are reasonably simple and scalable to large UWSNs, however they also suffer from serious drawbacks such as sparse network density, void communication regions, and inaccurate positioning of nodes, which also lead to excessive retransmissions, high energy consumptions and low packet delivery ratios. Therefore, one particular design goal of geographic information routing protocols for UWSNs is to minimize power consumption, while achieving high packet delivery ratio.

Ahlswede *et al.* [9] proposed a new information theory technique—network coding—to tackle the issues of power consumption and packet delivery ratio in multicast applications. Ever since its inception, a substantial number of researchers [10,11,12] have studied the benefits of network coding in wireless networks. Network coding allows each relay node to first encode received packets before forwarding the encoded data, which essentially decreases the size of transferred data, reduces the energy consumption at nodes, and improves the network bandwidth utilization, all contributing to the extension of the network lifetime. Network coding is also a promising technique for reducing data retransmissions and energy consumptions and for improving packet delivery ratio and network lifetime in UWSNs [13]. Because underwater sensor nodes possess more computational capabilities than those in wireless networks and furthermore the broadcast nature of underwater acoustic channels renders multiple routes from a source to a destination, the multiple routes coupled with the exceptional computational powers of the sensor nodes provide ample opportunities to apply network coding to geographic information routing in UWSNs.

**Figure 1:**
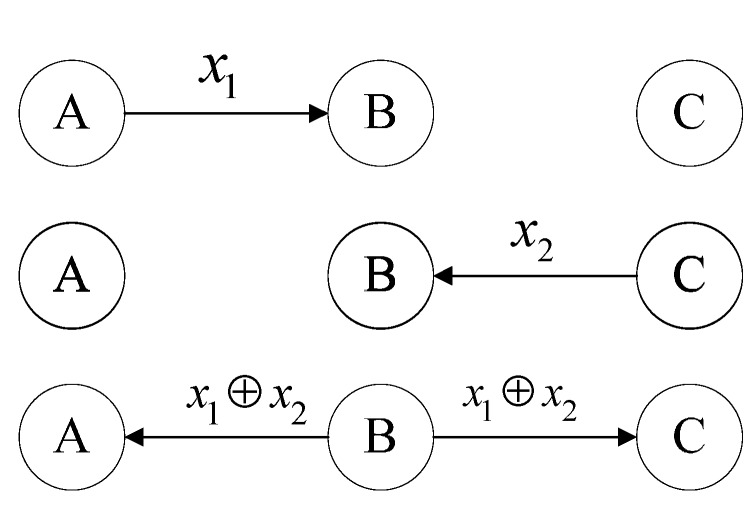
A network coding pattern.

Network coding can be illustrated by a well-known pattern shown in Figure 1, where two nodes A and C exchange data packets *x*_1_ and *x*_2_ via a relay node B. When node B receives packages *x*_1_ and *x*_2_ from nodes A and C respectively, it broadcasts $x_{1}⊕x_{2}$ (their binary XOR) to both nodes A and C. When node A receives $x_{1}⊕x_{2}$, it uses its knowledge of *x*_1_ to retrieve *x*_2_. In a similar spirit, when node C receives $x_{1}⊕x_{2}$, it uses its knowledge of *x*_2_ to retrieve *x*_1_.

However, with the traditional full network coding technique as illustrated by Figure 1, nodes in a network have to wait for the data packets from all other nodes to arrive before starting decoding, which inevitably increases the network delay. To tackle this issue, Wang *et al.* [14] proposed the partial network coding (PNC) solution that can effectively reduce the network delay. In this work, we propose GPNC*,* a novel geographic information routing protocol that adopts partial network coding for efficient forwarding of data packets through reducing retransmissions and network delays and improving data delivery rates. The GPNC protocol employs a novel forwarding strategy to route data packets to any destination node by choosing candidate forwarding nodes according to each node’s normalized packet advance and its residual energy in order to appropriately balance energy efficiency and data delivery rate. Simulation results show that GPNC can significantly improve network throughput and packet delivery ratio, while reducing energy consumption and network latency when compared with other routing protocols.

The rest of this paper is organized as follows: Section 2 reviews previous work on geographic information routing and network coding. Section 3 describes the network model and the partial networking coding model. Section 4 presents the GPNC routing algorithm, followed by its performance evaluation in Section 5. Finally, Section 6 concludes the paper with a summary of major contributions and future work.

## 2. Related Work

In this section, we review some of the important existing geographic information routing protocols and network coding schemes for UWSNs.

### 2.1. Geographic Information Routing

Geographic information routing uses the location information of sensor nodes to forward data packets from a source node to a destination node. Yan *et al.* [15] proposed the Depth Based Routing (DBR) protocol, which can extend the lifetime of an entire UWSN by addressing in the depth direction and performing routing adjustment based on depth difference. However, the DBR protocol is only suitable for relatively dense networks as the depth of two nodes are not significantly different in sparse networks.

Xie *et al.* [16] contributed the Vector-Based Forwarding (VBF) protocol, where during a routing process, each node does not need to save status information; instead it uses a forwarding factor to calculate the suppression time before forwarding is carried out in order to increase network energy efficiency by avoiding unnecessary forwarding, while the routing information is included in each data packet. However, the VBF protocol is also susceptible to network density, which impacts on the efficiency of creating a pipe from a source node to a destination node as there may be few nodes in a pipe for forwarding packages. In addition, the radius of pipe may significantly influence the routing performance. Nicolaou *et al.* [17] later presented the Hop by Hop VBF (HH-VBF) protocol to alleviate V*B*F’s problem of finding no forwarding node by creating a routing pipe for each forwarding node and by adopting redundant control in a self-adaption process. As a result, HH-VBF outperforms VBF in terms of both node energy consumption and data delivery rate.

The Focused Beam Routing Protocol (FBR) [18] is another protocol based on location information, which aims to reduce energy consumption in data transmission by restraining flooding of packets. It is suitable for both mobile and static UWSNs as it does not require synchronizing clocks at the sensor nodes. The Multi-Path Routing (MPR) protocol [19] solves the data collision problem at receiving nodes by preventing them from receiving packets from different relay nodes through constructing a routing path consisting of multiple subpaths between the source node and the destination node. Compared to both the VBF and HH-VBF protocols, the MPR protocol shows a higher throughput in dense networks but at the same time leads to a higher energy consumption as it uses a substantial number of matrix operations. HH-VBF shows a higher overhead as it relies on flooding to discover neighboring nodes.

Coutinho *et al.* [4] proposed the GEDAR routing protocol, which adopts geographic and opportunistic routing and uses depth adjustment based topology control for communication recovery over void regions. Greedy opportunistic forwarding is employed to route packets and move void nodes to new depths for the adjustment of topology. GEDAR outperforms the baseline solutions in terms of packet delivery ratio, but it exhibits high energy consumptions for low-density UWSNs.

### 2.2. Network Coding Schemes for Underwater Networks

Mo *et al.* [20] proposed the Practical Coding-based Multi-hop Reliable Date Transfer (PCMRDT) protocol to avoid sender-receiver and receiver-receiver collisions and to decrease overall average end-to-end delay by combining random linear coding and selective repeat. PCMRDT can significantly reduce the network delay while achieving a high energy efficiency. Chitre *et al.* [21] studied the problem of transmitting data efficiently in underwater sensor network. They compared the solutions based on Automatic Repeat Request (ARQ), network coding and erasure coding and found that the network coding based solution achieved a higher throughput than other solutions did.

Wu *et al.* [13] presented the Time Slot based Routing (TSR) algorithm, where network coding was used to further reduce the probability of node conflicts, decrease node energy consumption and extend network lifetime. Guo *et al.* [22] contributed a reliable underwater sensor routing algorithm VBF_NC based on network coding. They found that combining network coding and multi-path routing can achieve higher robustness in UWSNs. They compared their approach with single-path forwarding, multi-path forwarding, end-to-end Forward Error Correction (FEC) and even hop-by-hop FEC and proved that their approach was more efficient in terms of both error recovery and energy consumption.

These algorithms only use the error correction property of full network coding to improve the reliability of data transmission. However, nodes in a network have to wait for the data packets from all other nodes to arrive before starting decoding, which inevitably increases the network delay. To reduce the latency in data transmission, Hao *et al.* [23] proposed an opportunistic routing protocol based on partial network coding. In this work, we combine partial network coding and geographic routing to improve the performance of UWSNs. To the best of our knowledge, GPNC is the first geographic routing protocol based on partial network coding that is able to reduce the size of transferred data, the energy consumptions at nodes, and the network delays in data transmissions while improving the bandwidth utilization and the network lifetime.

## 3. Network and Partial Networking Coding Models

In this section, we present the conceptual models used by the proposed GPNC routing protocol. We first describe the underwater mobile sensor network model, which is then followed by the packet delivery probability model used by GPNC to select the next-hop forwarding set. Finally, we describe the partial network coding model used for transmitting data.

### 3.1. The Network Model

**Figure 2:**
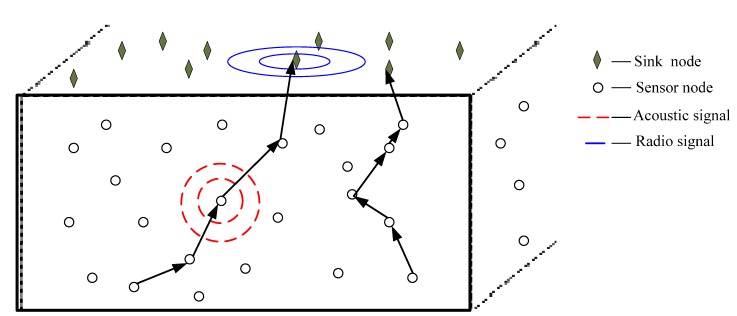
A 3D network structure.

Figure 2 depicts a three-dimensional network model used by the GPNC routing protocol, which includes several sink nodes and ordinary acoustic sensor nodes. All acoustic sensor nodes have the same structure and are randomly distributed in the water. Each sink node is equipped with a RF modern and an acoustic modern. We assume that each sensor node knows its 3D location information through location services and can save the location information about itself and about its destination node.

Sensor nodes are deployed under the water in a Euclidean space $D\in {ℜ}^{3}$. At any time *t*, we model the network as an undirected graph *G*(*t*) = (*V*,*ε*(*t*)) [4], where $V=\left\{n_{i}|1\leq i\leq M\right\}$ is the set of sensor nodes and $\varepsilon (t)=\left\{e_{ij}|1\leq i,j\leq M,i\neq j\right\}$ is the finite set of links between nodes at time *t*. For $\forall e_{ij}(t)\in \varepsilon (t)$, nodes *n_i_* and *n_j_* are neighbors at time *t* and can communicate directly with each other (send and receive messages) via an acoustic link. For $\forall n_{i}\in V$, the set of its neighbors at time *t* is defined as $N_{i}(t)=\{n_{i}\in V|\exists e_{ij}(t)\in \varepsilon (t)\}$. We assume that all nodes transmit data using the same transmitting power and with the same communication range *R*.

### 3.2. Packet Delivery Probability

During the initialization phase, each sink node broadcasts information including its transmission power *f* to all sensor nodes, which then each calculates its distance to the sink node based on the received signal strength. In the attenuation model of underwater acoustic signal, if the distance between a transmitting node and a receiving node is *x* and the geometry of signal propagation is described using spreading factor *k* (*k* = 1.5 for a practical scenario), then the attenuation factor is calculated by:

(1) $$A(x)=x^{k}a^{x}$$

where $a=10^{a(f)/10}$ and $a(f)=\frac{0.11f}{1+f^{2}}+\frac{44f^{2}}{4100+f^{2}}+2.75\times 10^{-4}f^{2}+0.003$.

Built on the foundation of a typical underwater acoustic channel model described by Equation (1), we use Rayleigh fading to model small-scale fading of signal propagation and Binary Phase Shift Keying (BPSK) to calculate the average Bit Error Rate (BER). If a symbol Signal-to-Noise ratio (SNR) is *r_s_* and a bit SNR is *r_b_*, then *r_s_* = *r_b_* if using BPSK. If we further define $\bar{r_{s}}=10^{r_{b}/10}$, then BER is calculated by:

(2) $$p_{b}(r_{b})=\frac{1}{2}(1-\sqrt{\frac{\bar{r_{s}}}{1+\bar{r_{s}}}})$$

The following passive sonar equation gives each bit SNR of the underwater signal at a receiving node:

(3) $$r_{b}=SL-k\times 10\times \log d-a(f)\times d\times 10^{-3}-50+18\times \log f$$

where *SL* (typically 118 dB) is the sound intensity level.

Finally, for any pair of nodes with the distance of *d*, the delivery probability of a packet with the size of *m* bits is given by:

(4) $$p(d,m)={\left(1-p_{b}(r_{b})\right)}^{m}$$

### 3.3. The Partial Network Coding Model

With partial network coding [14], each source node simply broadcasts the original data without encoding it. Each intermediate forwarding node can adopt an encoding method that uses an appropriate length to reduce the delay caused by waiting for the data packets from other nodes to arrive. Each destination node executes Gaussian digestion after receiving packets and decides whether decoding can be completed successfully. If decoding is done, each decoded packet is transmitted to the upper protocol layer; otherwise, the packet is inserted into the waiting queue. Partial network coding better adapts to dynamic networks than full network coding does, especially in terms of network delay, which can be illustrated by the following example, where the source node *S* transmits data packets *p*_1_, *p*_2_, *p*_3_, and *p*_4_ to node *D* via four intermediate nodes.

**Figure 3:**
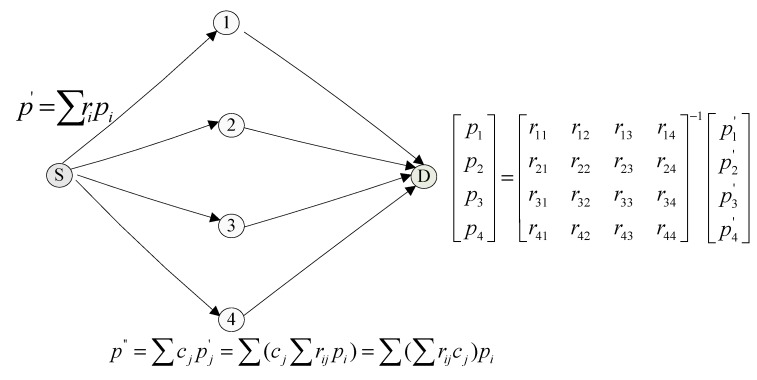
Full network coding algorithm.

The example in Figure 3 depicts a scenario of full network coding. The source node *S* encodes the data packets of *p*_1_ − *p*_4_ to be $p_{1}^{\prime }-p_{4}^{\prime }$ respectively and sends the encoded packets to the intermediate nodes, which in turn encode them again to be $p_{1}^{\prime \prime }-p_{4}^{\prime \prime }$ respectively and then forward the re-encoded packets to node *D*. Suppose packets $p_{1}^{\prime }$, $p_{2}^{\prime }$, $p_{3}^{\prime }$ and $p_{4}^{\prime }$ from node *S* arrive at node *D* at the time interval of *t*, that is their arrival times are *t*, *2t*, *3t*, and *4t* respectively. Node *D* can only decode the received packets back into the original data of *p*_1_, *p*_2_, *p*_3_, and *p*_4_ after all the four packets have been received and as a result the average delay of transmitting these data packets is (4*t* × 4)/4 = 4*t*.

**Figure 4:**
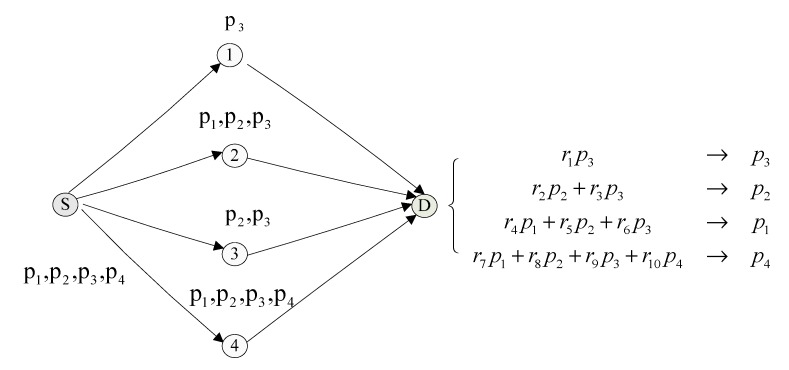
Partial network coding algorithm.

In contrast, Figure 4 shows an example of the partial network coding algorithm. The source node *S* broadcasts directly the original data packets to the intermediate nodes, which each encodes them using partial network coding of some length and then forwards the encoded packets to the node *D*. When node *D* receives the encoded data packet *r*_1_*p*_3_ forwarded by the intermediate node 1 at time *t*, it decodes it back to the original packet *p*_3_. When node *D* receives the encoded data packet *r*_2_*p*_2_ + *r*_3_*p*_3_ forwarded by the intermediate node 3 at time *2t*, it decodes *p*_2_ because *p*_3_ has already been decoded. In a similar token, when node *D* receives the encoded packets *r*_4_*p*_1_ + *r*_5_*p*_2_ + *r*_6_*p*_3_ and *r*_7_*p*_1_ + *r*_8_*p*_2_ + *r*_9_*p*_3_ + *r*_10_*p*_4_ from the nodes 2 and 4 at time *3t* and *4t* respectively, it can decode *p*_1_ and *p*_4_. So the average delay of transmitting the four packets is (*t* + 2*t* + 3*t* + 4*t*)/4 = 2.5*t*, which is much better than that of the full network coding algorithm.

**Figure 5:**
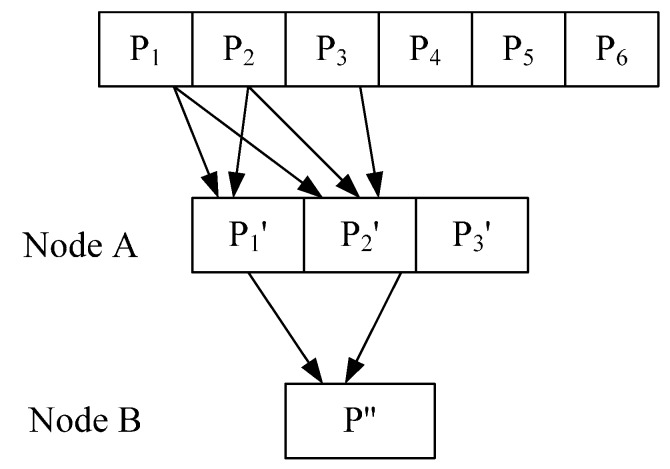
An intermediate node performing partial network coding.

The original data is divided into different blocks in partial network coding, each of which includes N packets. If the random selection strategy is adopted, an intermediate node then randomly selects several packets to encode from the cache of the same block. As shown in Figure 5, an intermediate node first generates a random integer M and then randomly selects M packets from the cache queue to encode before forwarding the encoded data packets.

Assume M = 2 and node A stores three packets from the same block: $p_{3}^{\prime }$, $p_{1}^{\prime }=r_{1}p_{1}+r_{2}p_{2}$, and $p_{2}^{\prime }=r_{1}^{\prime }p_{1}+r_{2}^{\prime }p_{2}+r_{3}^{\prime }p_{3}$. Then packets $p_{1}^{\prime }$ and $p_{2}^{\prime }$ are encoded by node B to be $p^{\prime \prime }=a_{1}p_{1}^{\prime }+a_{2}p_{2}^{\prime }=a_{1}(r_{1}p_{1}+r_{2}p_{2})+a_{2}(r_{1}^{\prime }p_{1}+r_{2}^{\prime }p_{2}+r_{3}^{\prime }p_{3})=(a_{1}r_{1}+a_{2}r_{1}^{\prime })p_{1}+(a_{1}r_{2}+a_{2}r_{2}^{\prime })p_{2}+a_{2}r_{3}^{\prime }p_{3}$.

With random partial network coding, the coefficients of the unselected data blocks at the intermediate nodes are likely to be zero and consequently the generated coding coefficient matrix at a receiving node is likely a sparse matrix. As it is easier to generate the inverse of a sparse matrix, the decoding rate at the receiving node is higher. If the matrix becomes full rank, the receiving node would be ready to perform decoding. But the coefficient matrix produced by random partial network coding is usually not full rank. As a result, the encoding vector would have a linear correlation and the receiving node would consequently need more than N encoded data blocks to perform decoding, which increases the transmission of invalid information and leads to more network consumption.

As the signal propagation speed in an underwater acoustic channel is very low, the delay in data transmission between nodes is significant in UWSNs. Furthermore, the error rate in data transmission is high, so some redundancy packets are required at a receiving node, which increases the actual energy consumption at intermediate nodes as it is proportional to the number of the forwarded packets. To tackle these issues, our GPNC protocol adopts partial network coding to reduce retransmissions, transmission delays, and energy consumptions. When an intermediate node receives an irrelevant linearly encoded packet, it knows it is a new packet and does not necessarily need to know what packet it is. Therefore, even if there is a packet loss, it is unnecessary to retransmit the lost packet, which greatly reduces the transmission of redundant data packets as well as energy consumption.

## 4. The GPNC Routing Algorithm

The proposed GPNC routing algorithm uses the geographic information on nodes for routing and partial network coding for data transmission. To forward a packet, it attempts to select a forwarding node that is nearest the destination node by considering the packet advancement. When a forwarding node needs to transmit data, it encodes the packet using partial network coding, and with the distance decreasing to the sink node, the encoded packet can finally arrive at the sink node. When the sink node has received N linear independently encoded data packets, it performs decoding to retrieve the packets.

The GPNC algorithm uses a greedy forwarding strategy to determine the set of next-hop forwarding nodes. Let *n_i_* be a source node that has a data packet to deliver, *N_i_*(*t*) be *n_i_*’s set of neighboring nodes at time *t*, and *S_i_*(*t*) be *n_i_*’s known set of sink nodes at time *t*. Considering a neighboring node $n_{i}\in N_{i}(t)$, its packet advancement is defined as [4]:

(5) $$ADV(n_{j})=D(n_{i},s_{i})-D(n_{j},s_{j})$$

where $D(n_{i},s_{i})$ is node *n_i_*’s Euclidean distance to its closest sink node $s_{i}\in S_{i}(t)$ at time *t* and $D(n_{j},s_{j})$ is node *n_i_*’s Euclidean distance to its closest sink node $s_{i}\in S_{i}(t)$ at time *t*.

Generally speaking, the greater the packet advancement is, the higher priority the neighboring node is given. Then *n_i_*’s set of next-hop forwarding nodes at time *t* is:

(6) $$C_{i}(t)=\{n_{j}\in N_{i}(t),ADV(n_{j}>0)\}$$

For a candidate forwarding node $n_{c}\in C_{i}$, $D(n_{i},n_{c})$ is the Euclidean distance between the source node *n_i_* and the forwarding node *n_c_*. Assume the packet delivery probability of *m* bits over distance $D(n_{i},n_{c})$ is $p(D(n_{i},n_{c}),m)$ as given by Equation (4). The candidate forwarding node *n_c_*’s normalized packet advancement is defined as:

(7) $$NADV(n_{c})=ADV(n_{c})\times p(D(n_{i},n_{c}),m)$$

Based on a node’s energy and NADV, we define the following weighting formula for ordering candidate forwarding nodes:

(8) $$W=\partial \frac{E_{r}}{E_{o}}+(1-\partial )NADV$$

where ∂ is an equivalence factor between a response node’s energy and NADV, *E_o_* and *E_r_* are the initial and residual energy of the response node respectively.

We use Equation (8) to calculate the weight of each candidate forwarding node in $C_{i}(t)$ and create a sorted list $F_{i}(t)$ from $C_{i}(t)$ ordered by their weight from high to low. The first node in $F_{i}(t)$ will be chosen as the next-hop forwarding node to transmit a data packet and only it fails to do so, the next one in $F_{i}(t)$ would be chosen. The process continues in that order until the data has been successfully delivered. The *h*−*th* node in $F_{i}(t)$ will start to transmit data if none of the preceding nodes has successfully done that within time $T_{w}^{h}$ defined below:

(9) $$T_{w}^{h}=T_{d}+\sum_{j=1}^{h}D(n_{j},n_{j+1})/v+h\times T_{p}$$

where *v* is the speed of sound under water, *T_p_* is the packet processing time, and $T_{d}=(R_{c}-D(n_{i},n_{c}))/v$ is the propagation delay.

After the optimal next-hop node has been selected, the source node forwards the data packet to the selected forwarding node, which then encodes the packet and becomes the source node. The routing process continues as such until the data packet has been delivered to the sink node. When the sink node has received all the required linear independently encoded data, it starts decoding to retrieve the original data packets.

## 5. Performance Evaluation

Any proposed new protocol needs to be tested and validated against results from open sea experiments. While numerous testbed systems have been developed to allow for inexpensive small-scale testing, they still require abundant resources to prepare and perform the experiments. Therefore, simulators have been commonly adopted as an alternative approach to protocol testing [24]. However, many of the existing simulators are not all inclusive as they only focus either on the physical communication layer or the networking layers [24]. Furthermore, many of them use a generic underwater acoustic channel model and consequently the simulation results may not accurately reflect the real-world applications.

After a thorough investigation, we chose Aqua-Sim [4,25] to conduct a series of simulations. Aqua-Sim was developed based on NS-2, a popular network simulator, and has the ability to simulate UWSNs. Compared with other wireless network simulators, Aqua-Sim offers many distinctive features such as underpinning to discrete-event-driven networks [26], support for mobile and 3D networks, simulation of high-fidelity underwater acoustic channels, and implementation of a complete protocol stack.

We evaluated the performance of the GPNC protocol by comparing it with DBR [15] and VBF_NC [22] using Aqua-Sim as the routing protocol simulator. DBR greedily forwards data packets to any sink node on the sea surface without using any mechanism to transmit data at all. VBF_NC instead employs vector-based forwarding to determine the routes from a source node to a target sink node and uses full network coding to transmit data packets. We first compare GPNC with VBF_NC in terms of network delay and energy consumption and then compare GPNC with both DBR and VBF_NC in terms of packet delivery rate.

We randomly deployed 800 sensor nodes in a 3D region of 2000 m × 2000 m × 2000 m and 64 sink nodes in a sea surface region of 2000 m × 2000 m. The data transmission rate of the underwater acoustic modern is 2500 bps [27]. The acoustic signal propagation speed is 1500 m/s. The sensor nodes each has a transmission range of *R* = 250 *m*, an equivalence factor of ∂=0.5, an initial energy of *E_o_*= 100 J, and an energy consumption rate of 60 uJ/bit. There are 1000 original data packets generated at the source node and the size of each packet is 64 KB.

### 5.1. Effect of the Size of Encoding Information Block N on the Network Performance

#### 5.1.1. Network Delay

**Figure 6:**
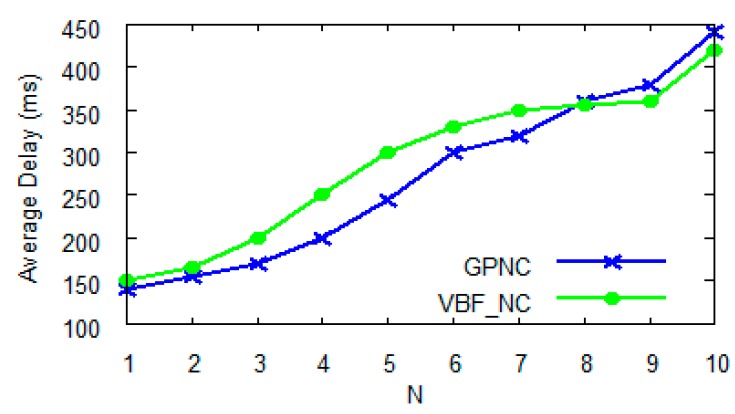
Impact of *N* on network delay.

**Figure 7:**
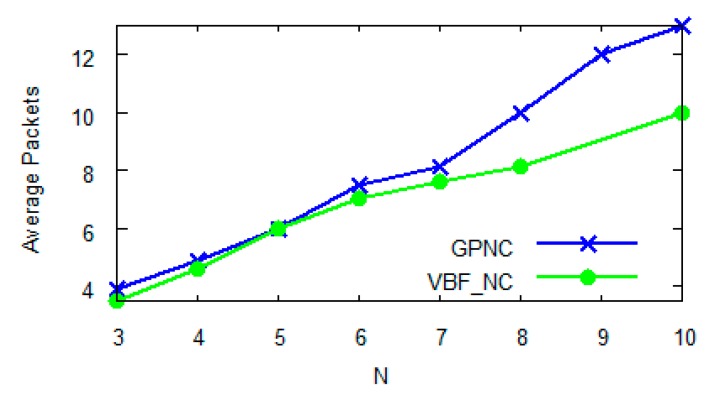
Impact of *N* on the number of required packets for decoding.

With (full or partial) network coding, the value of *N*, which is the number of packets included in each data block, impacts the time (network delay) of transmitting the data. We first conducted several experiments to compare GPNC (based on partial network coding) and VBF_NC (based on full network coding) in terms of network delay impacted by the selection of *N*. As shown in Figure 6, with the increase of *N*, network delay increases for both GPNC and VBF_NC. The average delay of GPNC is smaller than that of VBF_NC when *N* < 8, and especially the improvement is about 22% when 3 ≤ *N* ≤ 5. When *N* > 5, improvement of delay effect is not obvious and when *N* > 8, the delay of GPNC even exceeds that of VBF_NC. The longer delay for *N* > 5 is caused by the increase of the number of data packets to be decoded. Figure 7 further explains this phenomenon.

Normally, VBF-NC requires *N* encoded data packets to decode them back to the *N* original data packets, or the possibility of linear dependency is very small, which never exceeds *N* + 2. When *N* < 5, the number of required data packets for decoding in GPNC is similar to that in VBF-NC. However when *N* > 8, the number of required data packets for decoding in GPNC will exceed that in VBF-NC, leading to the increase of network delay and the decrease of network performance. The reason behind this is the essential difference between partial and full network coding algorithms. The partial network coding algorithm randomly selects packets for encoding when forwarding data. As a result, the same packets may be repeatedly encoded when *N* gets bigger, reducing the linear independency between packets and increasing the number of packets to be decoded and consequently the network delay. In contrast, the full network coding algorithm always maintains the linear independency between packets such that decoding can only be done after the *N* linearly independent packets have all been received by a receiving node.

#### 5.1.2. Network Throughput

**Figure 8:**
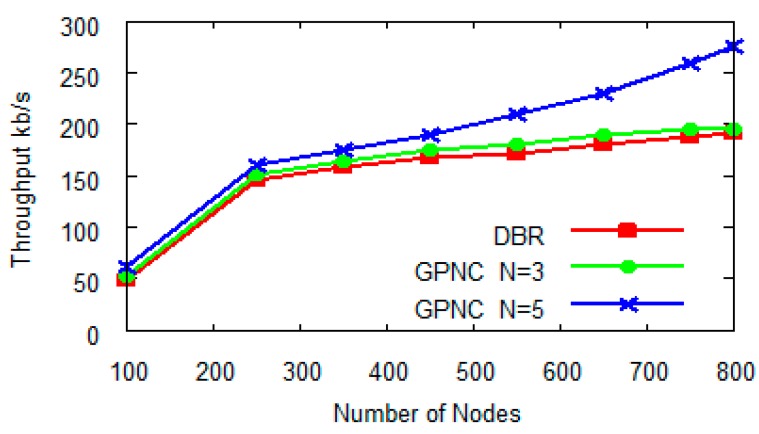
Throughput with number of nodes.

Throughput is another measure of network performance. A protocol with network encoding is expected to excel in throughput when compared to one without network coding, subject to the choice of *N*. Figure 8 compares the throughputs of DBR (without network coding), GPNC (*N* = 3) and GPNC (*N* = 5). With the increasing number of network nodes, throughput increases and gradually levels off for all three protocols, which is mainly because the increase of the overall network throughput is restrained by the competition among nodes. Figure 8 further reveals that the choice of *N* in GPNC has a clear impact on network throughput. When *N* = 3, the throughput of GPNC is similar to that of DBR because its opportunity of encoding packets is low. When *N* = 5, the throughput of GPNC is 22% better than that of DBR and the advantage of network coding is obvious. In conclusion, as *N* = 5 yields an optimal network performance shown in Figure 6 and Figure 8, we choose *N* = 5 in later experiments.

### 5.2. Energy Consumption

**Figure 9:**
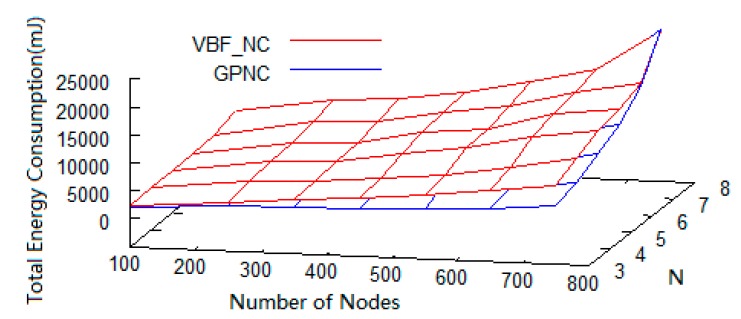
Energy consumption with the number of nodes and the value of *N*.

**Figure 10:**
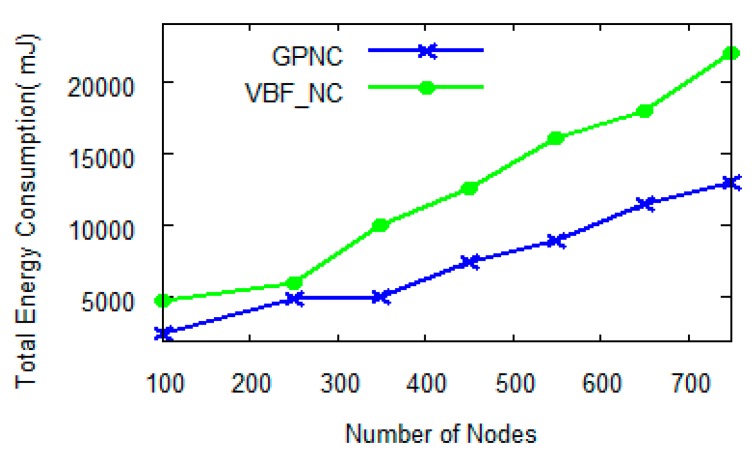
Energy consumption with the number of nodes when *N* = 5.

With network coding, the energy consumption in a UWSN increases with the number of sensor nodes as well as the value of *N*. However, as shown in Figure 9, when *N* ≤ 6, the growth curve of GPNC is clearly lower than that of VBF_NC thanks to the fact that GPNC considers node energy consumption when selecting a path. GPNC avoids using low-energy nodes for forwarding packets in order to assure the transmission reliability and increase the lifetime of the network nodes. In particular, Figure 10 shows the difference of energy consumption between GPNC and VBF_NC when *N* = 5.

### 5.3. Packet Delivery Ratio

**Figure 11:**
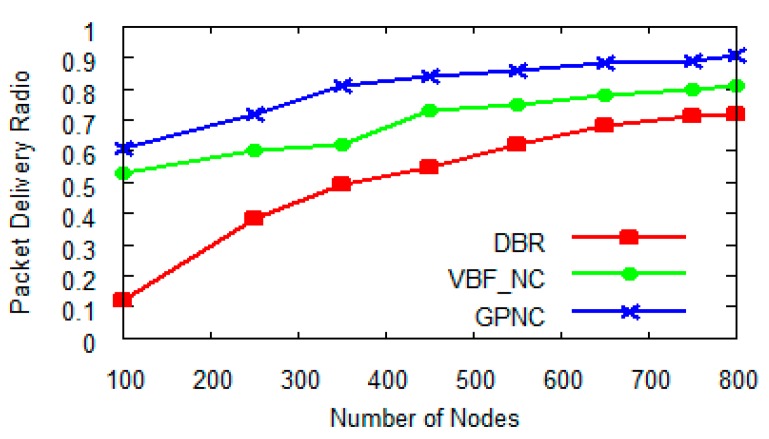
Packet delivery ratio with the number of nodes when *N* = 5.

We then conducted a number of experiments to compare the packet delivery ratio of GPNC with those of DBR and VBF_NC in terms of the number of nodes and the loss rate. Generally speaking, the packet delivery ratio increases with the number of nodes for all the three protocols, however, as shown in Figure 11, the protocols using network coding, which are GPNC and VBF_NC, achieve higher delivery ratios than the DBR protocol, which does not use network coding at all. Network coding can increase delivery rate because it can not only decrease the number of sending packets but also reduce collision and competition between packets. More importantly, GPNC’s delivery ratio is about 16% higher than that of VBF_NC when *N* = 5 (GPNC’s network delay is 22% lower than that of VBF_NC as shown in Figure 6).

**Figure 12:**
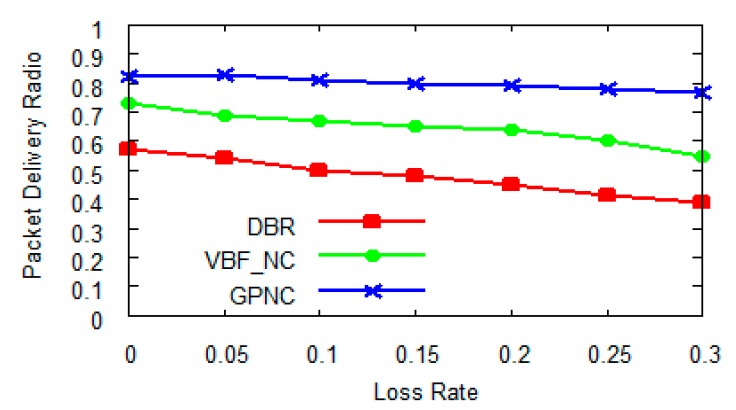
Packet delivery ratio with the increase of loss rate when *N* = 5.

Figure 12 further shows that the packet delivery ratio decreases with the increase of packet loss rate of transmission link for all the three protocols. However, the protocols using network coding, which are GPNC and VBF_NC, again achieve much higher delivery ratios than the DBR protocol (GPNC and VBF_NC are 45% and 26% better than DBR respectively) because with network coding, data packets do not need to be retransmitted even if some data is lost in transmission. More importantly, GPNC’s delivery ratio is about 19% higher than that of VBF_NC as GPNC employs multiple sink nodes for efficient data transmission.

## 6. Conclusions and Future Work

In this paper, we have presented a novel geographic and partial network coding based routing protocol for UWSNs called GPNC. GPNC uses partial network coding for data delivery in order to decrease the number of sending packets and reduce collision between packets. GPNC can improve the network delivery rate and at the same time reduce the energy consumption and network delay. GPNC uses a new greedy approach to deliver encoded packets to the sink nodes and chooses candidate nodes according to a node’s normalized packet advance (NADV) and residual energy. Simulation results have shown that GPNC improves network throughput and the data delivery ratio, while reducing energy consumption and network delay when compared with the baseline routing protocols.

How to avoid “void” areas is very important for any greedy strategy, so we plan to investigate how the depth adjustment of some nodes can impact void areas and how opportunity forwarding of data packets to sink nodes can be incorporated into the GPNC routing protocol.
